# Identification of novel host biomarkers in plasma as candidates for the immunodiagnosis of tuberculosis disease and monitoring of tuberculosis treatment response

**DOI:** 10.18632/oncotarget.11420

**Published:** 2016-08-19

**Authors:** Ruschca Jacobs, Stephanus Malherbe, Andre G. Loxton, Kim Stanley, Gian van der Spuy, Gerhard Walzl, Novel N. Chegou

**Affiliations:** ^1^ Department of Biomedical Sciences, DST/NRF Centre of Excellence for Biomedical Tuberculosis Research and SAMRC Centre for Tuberculosis Research, Division of Molecular Biology and Human Genetics, Faculty of Medicine and Health Sciences, Stellenbosch University, Cape Town, South Africa

**Keywords:** tuberculosis, diagnosis, biomarker, neural cell adhesion molecule (NCAM), acute phase proteins, Immunology and Microbiology Section, Immune response, Immunity

## Abstract

There is an urgent need for new tools for the rapid diagnosis of tuberculosis disease. We evaluated the potentials of 74 host markers as biomarkers for the immunological diagnosis of tuberculosis and monitoring of treatment response. Fifty-five individuals that presented with signs and symptoms requiring investigation for tuberculosis disease were prospectively recruited prior to clinical diagnosis, at a health centre in Cape Town, South Africa. Patients were later classified as having tuberculosis disease or other respiratory diseases (ORD) using a combination of clinical, radiological and laboratory findings. Out of 74 host markers that were evaluated in plasma samples from study participants using a multiplex platform, 18 showed potential as tuberculosis diagnostic candidates with the most promising being NCAM, CRP, SAP, IP-10, ferritin, TPA, I-309, and MIG, which diagnosed tuberculosis disease individually, with area under the ROC curve ≥0.80. Six-marker biosignatures containing NCAM diagnosed tuberculosis disease with a sensitivity of 100% (95%CI, 86.3-100%) and specificity of 89.3% (95%CI, 67.6-97.3%) irrespective of HIV status, and 100% accuracy in the absence of HIV infection. Furthermore, the concentrations of 11 of these proteins changed with treatment, thereby indicating that they may be useful in monitoring of the response to tuberculosis treatment. Our findings have potential to be translated into a point-of-care screening test for tuberculosis, after future validation studies.

## INTRODUCTION

Tuberculosis (TB) disease, although curable, still accounted for the deaths of 1.5 million people in 2014 [1]. Rapid and accurate tools are urgently needed for early diagnosis of the disease, and monitoring of the response to treatment. The gold standard test for TB (culture) is not widely available, especially in resource-poor settings. The Ziehl Nielsen sputum smear test is often the only available diagnostic tool in these settings, even though its limitations are well publicised [2]. Month 2 culture conversion is the most investigated biomarker for TB treatment response, but the wide unavailability of culture and its long turn-around time are serious limitations. Smear microscopy is not very useful for monitoring anti-TB treatment response as it is unable to distinguish live from dead bacilli [3]. The development of the geneXpert MTB/RIF test (Cepheid Inc., Sunnyvale, USA) was a significant advance in the TB diagnostic field as the test yields results within 2 hours, coupled with the detection of resistance to rifampicin, as a proxy for multi-drug resistant TB [4]. The high operating costs and need for infrastructure are amongst the major obstacles for its implementation in resource-poor settings. The geneXpert test is also not useful in monitoring of TB treatment response as the test cannot distinguish between DNA from dead and live bacteria [5]. Immunodiagnostic techniques may be useful in both the diagnosis of TB disease and monitoring of the response to treatment, especially as they may be easily adaptable into rapid, point-of-care tests, which would be suitable in resource-constrained settings. Furthermore, such tests will also be beneficial in cases where a conventional sputum-based diagnosis (smear microscopy, culture or geneXpert) is difficult e.g. in paediatric TB and in individuals with extrapulmonary TB.

IFN-gamma (IFN-γ) release assays (IGRA) remain the most widely used commercial immunodiagnostic tests for TB. These assays have been shown to be useful in the diagnosis of infection with *Mycobacterium tuberculosis (MTB)* but as they cannot discriminate between active TB disease and latent *MTB* infection, they are of limited value in high TB-endemic areas. The use of IGRAs as tools for monitoring of the response to TB treatment has so far yielded conflicting results [6] [7]. An important limitation of overnight culture-based assays such as IGRAs is the fact that they are not suitable as point-of-care tests. These tests are therefore not ideal for resource-constrained settings. The potential value of diagnostic approaches that are based on the detection of host biomarkers *ex vivo*, in easily obtainable samples such as saliva, serum or plasma has been demonstrated in previous studies [8] [9] [10] [11]. Such host biomarker based tests in addition to being used as diagnostic tests for TB, may also be useful in monitoring of the response to TB treatment [8] [12] [13]. Despite the promise so far shown in these previous investigations, no validated diagnostic tests based on the detection of host biomarkers in unstimulated samples currently exist. It is therefore important to validate the potential biomarkers that have so far been identified, and also identify new candidate host markers that might be useful in conjunction with previously identified biosignatures, in the diagnosis of TB disease.

In the present study, we investigated multiple host markers, including previously identified promising host markers, and relatively new markers, some of which have not previously been investigated in the TB field, as candidates for the immunological diagnosis of TB disease and monitoring of the response to TB treatment.

## RESULTS

A total of 55 study participants, 22 of whom were culture positive TB patients were investigated in this study. The mean age of all study participants was 35.8 ± 10.2 years and 14 (25%) were HIV infected. The clinical and demographic characteristics of study participants are shown in Table 1.

**Table 1:** Clinical and demographic characteristics of study participants

Number of participants	All (*n* = 55)	TB (*n* = 22)	ORD (*n* = 33)
Males, *n* (%)	22 (40)	7 (32)	15 (45)
Mean age, (Years)±SD	35.8 ± 10.2	38.8 ±10.1	33.9 ± 9.9
HIV Infected, n(%)	14(25)	4(18)	10(30)
Quantiferon results			
Positive, *n* (%)	34 (64)	15 (75)	19 (58)
Negative, *n* (%)	18 (34)	4 (20)	14 (42)
Indeterminate, *n* (%)	1 (2)	1 (5)	0 (0)

All the 22 TB patients included in the study were culture positive

Abbreviations: TB= pulmonary tuberculosis, SD=standard deviation

### Utility of individual host markers in the diagnosis of TB disease

**Figure 1:**
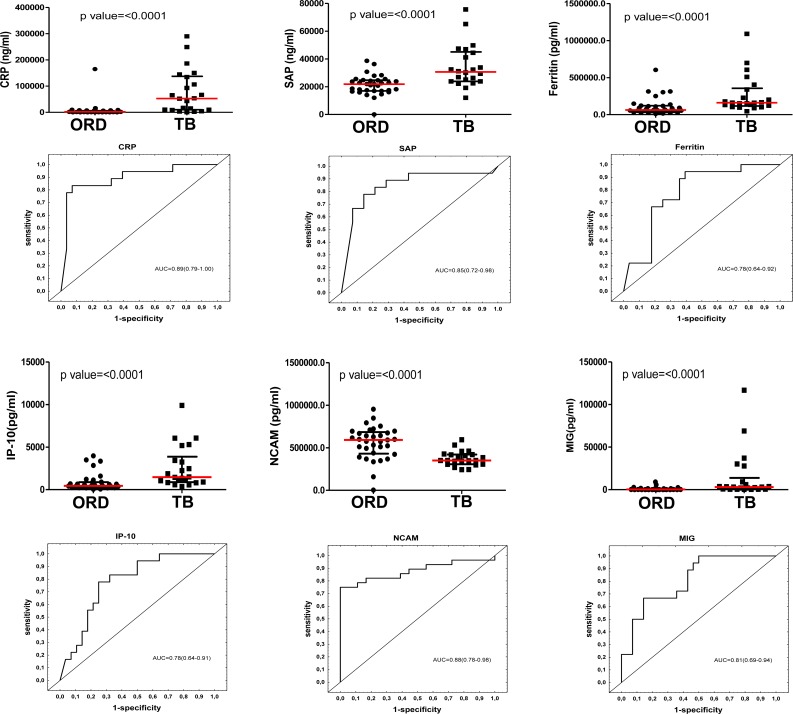
Concentrations of host markers detected in plasma samples from TB patients (*n* = 22) and individuals with other respiratory diseases (*n* = 33) and receiver operator characteristics curves showing the accuracies of these markers in the diagnosis of TB disease Representative plots are shown for CRP, SAP, ferritin, IP-10, NCAM and MIG. Error bars in the scatter dot plots represent the median with interquartile range.

When the baseline concentrations of host markers in TB patients (*n* = 22) were compared to the levels detected in patients with ORD (*n* = 33), by the Mann Whitney U test, the concentrations of 23 out of the 74 analytes were significantly different between the two groups. The median levels of CRP, SAP, PCT, ferritin, TPA, SAA, ADAMTS-13, p-selectin, GDF-15, I-309, IFN-γ, IP-10, TNF-α, CFH, MIG, ITAC, HCC-1 and MIP-4 were significantly higher in TB cases, whereas the levels of antithrombin III, Apo A-1, transthyretin, NCAM and BDNF were significantly higher in the ORD group. Trends (0.05 < *p* ≤ 0.01) towards higher levels of sFas, lipocalin-2, VEGF, PEDF, CC4 and IL-33 were observed in TB cases (Table 2). When the diagnostic accuracies of individual host markers were investigated by ROC curve analysis, the area under the ROC curve (AUC) was ≥ 0.70 for 18 markers (Table 2). The most accurate single host markers included CRP, SAP, NCAM, TPA, I-309, and MIG, which all performed with AUC ≥ 0.80 (Table 2). Representative plots showing some of the most accurate individual host markers are shown in Figure 1. When data was stratified according to HIV infection status, concentrations of three additional markers (A2M, MIP-1β and VEGF) became significant in the two groups, with AUC's of 0.70, 0.69 and 0.69 respectively.

**Table 2:** Median levels (and inter-quartile ranges in parenthesis) of host biomarkers detected in baseline plasma samples from pulmonary TB patients (*n* = 22) and individuals with other respiratory diseases (*n* = 33) and their diagnostic accuracies for TB disease

Marker	ORD (*n* = 33)	TB Disease (*n* = 22)	*P* value	AUC (95% CI)	Cut-off value	Sensitivity % (95% CI)	Specificity % (95% CI)
**ADAMTS-13**	3297(2569-4504)	4235 (2766-8073)	0.044	0.66 (0.51-0.81)	> 3466	68 (45-86)	64 (45-80)
**Antithrombin III**	803100 (624200-968800)	625700 (519000-714300)	0.01	0.70 (0.56-0.84)	<744162	91 (71-99)	61 (42-77)
**Apo A-1**	431400 (334100-548700)	274300 (242800-351500)	0.0014	0.76 (0.62-0.89)	< 318930	73 (50-89)	82 (65-93)
**BDNF**	5774 (3824-8838)	3791 (1683-6187)	0.017	0.69 (0.55-0.84)	< 3467	45 (24-68)	91 (76-98)
**CC4**	114800 (70110-176600)	151600 (95100-291100)	0.100	0.63 (0.48-0.79)	> 212263	32 (14-55)	97 (84-100)
**CFH**	729100 (557000-795100)	875400 (715200-980300)	0.0072	0.72 (0.57-0.86)	> 808359	68 (45-86)	82 (65-93)
**CRP**	2019 (440-6330)	52980 (10020-137400)	*P*<0.0001	0.89 (0.79 −1.00)	> 9081	82 (60-95)	90 (76-98)
**Ferritin**	62850 (41840-120100)	161000 (116800-355300)	*P*<0.0001	0.78 (0.64 −0.92)	> 93785	91 (71-99)	67 (48-82)
**GDF-15**	19.2(9.8-41.7)	49.24 (25.10-125.5)	0.002	0.75 (0.62 −0.88)	> 21.06	91 (71-99)	55 (36-72)
**HCC1**	108000 (73120-130200)	144100 (108500-171800)	0.0022	0.75 (0.61-0.89)	> 136956	59 (36-79)	85 (68-95)
**I-309**	1.24(1.1-1.4)	2.25 (1.4-3.5)	0.0002	0.80 (0.67-0.93)	> 1.945	68 (45-86)	90 (73-98)
**IFN-γ**	5.78(0.39-49)	31.06 (8.81-156)	0.02	0.69 (0.54 −0.83)	> 3.910	91 (70-99)	48 (31-66)
**IL-33**	88.77 (21.75-211.5)	164.9 (70.79-251.9)	0.100	0.63 (0.48-0.78)	> 131.8	68 (45-86)	61 (42-77)
**IP-10**	444 (258-876)	1469 (878-3865)	P<0.0001	0.78 (0.64 −0.91)	> 746.6	86 (65-97)	73 (54-87)
**ITAC**	628.0 (87.49-1253)	1106 (519.1-2042)	0.022	0.68 (0.54-0.83)	> 276.5	95 (77-100)	36 (20-55)
**Lipocalin-2**	453.9 (300.5-567.3)	600.7 (346.7-1028)	0.062	0.65 (0.50-0.80)	> 552.8	59 (36-79)	76 (58-89)
**MIG**	312.4 (87.21-1028)	3076 (592.2-13830)	*P*<0.0001	0.81 (0.69-0.94)	> 1700	68 (45-86)	88 (72-97)
**MIP-4**	92.5 (53.6-152)	208 (90-369)	0.012	0.70 (0.55-0.85)	> 220.9	50 (28-72)	91 (76-98)
**NCAM**	592100 (430200-684200)	350800 (306800-421000)	*P*<0.0001	0.88 (0.78-0.98)	< 477229	91 (71-99)	73 (54-87)
**PCT**	7520 (6749-8370)	8702(8185-9888)	0.0009	0.77 (0.64-0.90)	> 8101	86 (65-97)	67 (48-82)
**PEDF**	10790 (8852-12870)	12360 (10360-15270)	0.0502	0.66 (0.50-0.81)	> 11423	68 (45-86)	64 (45-80)
**p-selectin**	202(163-549)	441 (263-796)	0.030	0.67 (0.53 −0.82)	> 265.7	77 (55-92)	58 (39-75)
**SAA**	5972(1324-12570)	9837 (6078-43000)	0.0081	0.71 (0.58 −0.85)	> 8626	68 (45-86)	70 (51-84)
**SAP**	21850 (16980-24670)	30660 (23820-45050)	*P*<0.0001	0.85 (0.72-0.98)	> 25958	68 (45-86)	85 (68-95)
**sFAS**	5.3 (1.8-8.0)	8.03 (4.52-13.22)	0.065	0.65 (0.50-0.81)	>6.7	67 (43-85)	68 (49-83)
**TNF-a**	7.4(4.1-13.4)	15.9 (11.8-24.6)	0.0024	0.74 (0.61-0.88)	> 10.85	82 (60-95)	73 (54-87)
**TPA**	5895 (5187-6507)	7199 (6536-7702)	0.0002	0.80 (0.68-0.92)	> 6307	86 (65-97)	76 (58-89)
**Transthyretin**	544700 (398000-638500)	293700 (212700-397500)	0.0005	0.78 (0.65-0.91)	< 416242	82 (60-95)	76 (58-89)
**VEGF**	147 (0-546)	289 (134.3-877)	0.081	0.64 (0.50-0.79)	> 175.6	73 (50-89)	55 (36-72)

Only analytes showing significant differences or trends between groups with the Mann-Whitney U test are shown. Optimal cut-off values and associated sensitivity and specificity were determined based on the Youden's Index. The concentrations of CRP, SAP, SAA, antithrombin III, ADAMTS-13, p-selectin, GDF-15, Apo A-1, transthyretin, CFH, sFAS, lipocalin-2, MIP-4 and CC4 are in ng/ml. The concentrations of all the other analytes are in pg/ml.

### Utility of multi-plasma marker biosignatures in the diagnosis of TB disease

**Figure 2:**
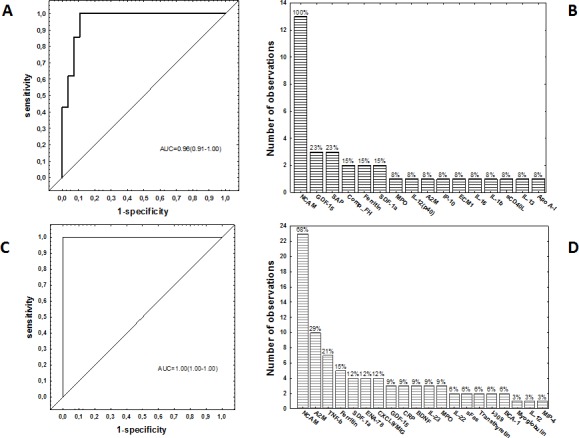
Accuracy of multi-marker models in the diagnosis of TB disease Receiver operator characteristics (ROC) curve showing the accuracy of the most accurate six-marker biosignature (NCAM, SAP, IL-1β, sCD40L, IL-13 and Apo A-1) in the diagnosis of TB disease in all study participants, regardless of HIV infection status **A.**, frequency of analytes in the top 13 general discriminant analysis (GDA) models that most accurately classified study participants as TB disease or ORD irrespective of HIV status **B.**, ROC curve showing the accuracy of the most accurate six-marker biosignature (NCAM+A2M+IL-22+ferritin+ myoglobulin+IL-12(p40) or NCAM+A2M+IL-22+ferritin+TNF-β+MIP-4) in the diagnosis of TB disease in HIV negative study participants **C.**, and frequency of analytes in the top 34 GDA models that most accurately classified study participants as TB disease or ORD in the absence of HIV infection **D.**. The bar graphs **B.** and **D.** indicate the frequency of analytes in the most accurate GDA models.

When the data obtained from all the TB patients and those with ORD were fitted into General Discriminant Analysis (GDA) models regardless of HIV status, combinations between up to six different host markers showed potential in the diagnosis of TB disease. A five-marker biosignature comprising of NCAM, SAP, ferritin, CFH and ECM-1 diagnosed TB disease with a sensitivity of 95.2% (95% CI, 81.0-99.9%) and specificity of 92.9% (95% CI, 70.8-98.9%) in the resubstitution classification matrix and sensitivity of 95.2% (95% CI, 81.0-99.9%) and specificity of 89.3% (95% CI, 66.4-97.2%) after leave-one-out cross validation. However, the most optimal diagnostic biosignature irrespective of HIV status was a combination between six markers (NCAM, SAP, IL-1β, sCD40L, IL-13 and Apo A-1), which diagnosed TB disease with a sensitivity 100% (95% CI, 86.3-100%) and specificity of 89.3% (95%CI, 67.6-97.3%) after leave-one-out cross validation. The positive and negative predictive values of the six-marker biosignature were 87.5% (95% CI, 66.5-96.7%) and 100% (95%CI, 83.4-100%) respectively (Figure 2, Table 3).

When the GDA procedure was repeated after excluding the HIV infected individuals, two six-marker biosignatures:- NCAM, A2M, IL-22, ferritin, myoglobulin and IL-12(p40), and NCAM, A2M, IL-22, ferritin, TNF-β and MIP-4, diagnosed TB disease with both sensitivity and specificity of 100% (AUC = 1.0, 95% CI, 1.0-1.0) (Table 3). NCAM was the most frequent analyte in biosignatures, appearing in all the top 13 biosignatures for diagnosing TB disease regardless of HIV infection status, and in 68%(23 of the 34) biosignatures that were generated for the diagnosis of TB disease after excluding HIV infected individuals. Other markers that occurred most frequently in diagnostic biosignatures for TB disease included GDF-15, SAP, CFH, A2M, TNF-β, ferritin, SDF-1α amongst others (Figure 2).

**Table 3:** Accuracies of plasma protein biosignatures in the diagnosis of TB disease

Biosignature	Resubstitution Classification matrix					Leave-one-out cross validation			
	Sensitivity % (95% CI)	Specificity % (95%, CI)	PPV % (95% CI)		NPV % (95% CI)	Sensitivity % (95% CI)	Specificity % (95% CI)	PPV % (95% CI)	NPV % (95% CI)
***Accuracy of biosignatures regardless of HIV infection status***									
NCAM+ SAP+ ferritin+ CFH+ECM-1	95.2 (81.0-99.9)	92.9 (70.8-98.9)		90.9 (69.4-98.4)	96.3 (79.1-99.8)	95.2 (81-99.9)	89.3 (66.4-97.2)	87 (65.3-96.6)	96.2 (78.4-99.8)
NCAM+ SAP+IL-1β+sCD40L +IL-13+Apo A-1	100 (86.3-100)	89.3 (67.6-97.3)		87.5 (66.5-96.7)	100 (83.4-100)	100 (86.3-100)	89.3 (67.6-97.3)	87.5 (66.5-96.7)	100 (83.4-100)
***Accuracy of biosignatures in HIV uninfected individuals***									
NCAM+A2M+IL22+ ferritin+ myoglobulin+IL-12(p40)	100 (78.1-100)	100 (79.1-100)		100 (78.1-100)	100 (79.1-100)	100 (78.1-100)	100 (79.1-100)	100 (78.1-100)	100 (79.1-100)
NCAM+A2M+IL22+ ferritin+ TNF-β+MIP-4	100 (78.1-100)	100 (79.1-100)		100 (78.1-100)	100 (79.1-100)	100 (78.1-100)	100 (79.1-100)	100 (78.1-100)	100 (79.1-100)

Only the top two biosignatures generated for the diagnosis of TB disease, regardless of HIV infection status and in the HIV uninfected individuals only, are shown. The importance of the different analytes in biosignatures for the diagnosis of TB disease is shown in Figure 2.

### Changes in host biomarker levels during the course of TB treatment

**Figure 3:**
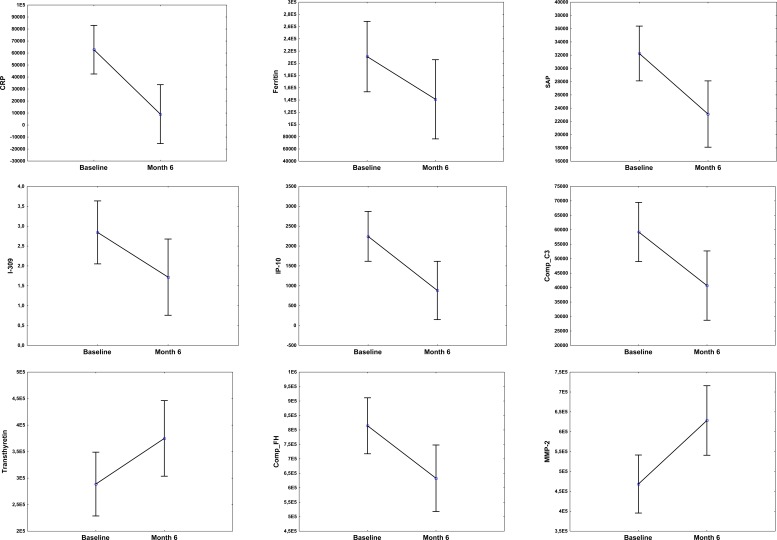
Before (baseline) and after treatment (month 6) concentrations of host markers in plasma samples from TB patients Plasma was collected from patients at recruitment, prior to the initiation of anti-TB therapy and then at the end of standard TB treatment (month 6). Error bars indicate the Least Squared means with 95% Confidence Intervals.

To investigate whether any of the 74 host markers could potentially be used to monitor the response to TB treatment, the host markers were evaluated in plasma samples that were collected from TB patients at the end of standard TB treatment (month 6). Of the 22 TB patients that were investigated in this study however, only 15 (68%) returned to the clinic and provided samples at the end of treatment. Compared to baseline levels, the concentrations of 11 host markers changed significantly during the course of treatment. There was a significant decrease in the levels of CRP, SAP, ferritin, IFN-γ, VEGF, IP-10, CC3, CFH and α-1-antitrypsin from baseline to month 6, whereas a significant increase in the levels of transthyretin and MMP-2 was observed. The levels of IL-1β, SAA, sFas and MIG showed trends towards decreasing levels from baseline to month 6, whereas Apo-CIII, Apo A-1 and GCP-2 showed trends towards increasing levels at the end of treatment (Figure 3).

## DISCUSSION

We investigated the diagnostic potentials of 74 host markers in plasma samples that were obtained from confirmed active TB cases and individuals with ORD, as candidates for the diagnosis of TB disease. Although 18 of the 74 host markers including relatively new biomarkers in the TB field namely; antithrombin III, GDF-15, NCAM, HCC1, MIP-4 and recently identified markers I-309, MIG, Apo A-1, transthyretin and CFH, showed potential in the diagnosis of TB disease, regardless of HIV infection status as determined by area under the ROC curve (AUC), the most optimal diagnostic biosignature irrespective of HIV infection status was a six-marker model comprising of NCAM, SAP, IL-1β, sCD40L, IL-13 and Apo A-1, which diagnosed TB disease with a sensitivity of 100% and specificity of 89.3%, with promising positive and negative predictive values. In the absence of HIV infection, six-marker biosignatures diagnosed TB disease with 100% accuracy.

Amongst the 18 host markers that showed the most potential in the diagnosis of TB disease individually as determined by AUC, six (CRP, SAP, PCT, ferritin, TPA and SAA) were acute phase proteins, six (I-309, MIG, MIP-4, Apo A-1, transthyretin and CFH) were markers that play various roles in the body and have recently been identified as promising TB diagnostic candidates [16] [11], two (TNF-α and IP-10) are widely investigated TB biomarkers [17] whereas four (antithrombin III, GDF-15, NCAM, HCC1), were relatively new markers, which have not previously been investigated in the TB field. Other host markers including ECM-1, IL-1β, sCD40L and IL-13 although not very promising individually in the current study, were included into the top diagnostic biosignatures for TB disease.

Acute phase proteins are primarily produced by the liver, and act as opsonins at inflammatory sites [18]. Serum CRP has been extensively studied and shown to be promising for both the diagnosis of TB disease and monitoring of TB treatment response [19] [20]. SAP, a homologue of CRP, has also been shown to have a protective role against bacterial infections [21]. Ferritin is a well-recognised protein in iron storage processes. It has long been established that iron acquisition is an essential virulence mechanism for pathogenic bacteria [22] [23]. The concentrations of all the acute phase proteins that showed potential in the current study were elevated in the TB patients, and this is in agreement with previous findings [23] [11] [24].

The chemokines; MIG(CXCL9), IP-10(CXCL10), ITAC(CXCL11) and I-309(CCL-1) are found in abundance in activated bronchial epithelium [25]. MIP-4(CCL18) is a chemokine that is produced mainly by antigen presenting cells [26]. These chemokines play vital roles in the recruitment of activated T-cells to the site of infection [25] [27]. Our finding of significantly higher levels of these chemokines in the plasma of patients with active TB is in agreement with previous observations [16] [28] [29] [26]. The relatively new host markers investigated in the study (GDF-15, antithrombin III, HCC1 and NCAM) showed potential individually, in the diagnosis of TB disease. Other markers including p-selectin, ADAMTS-13 and BDNF although not amongst the most promising single markers as demonstrated by ROC curve analysis, were significantly different between the TB patients and individuals with ORD, with the Mann-Whitney U test. GDF-15 is a member of the transforming growth factor beta superfamily (TGF-β) and its expression is associated with tissue damage, but has also been reported to exhibit tissue protective functions [30] [31]. It has been identified as a prognostic marker for prostate cancer, with high serum levels observed in patients with liver cirrhosis and hepatocellular carcinoma [32] [33]. Antithrombin complexes are important mediators of the coagulation system, with antithrombin III being one of the most important inhibitors of this system [34]. Markedly lowered antithrombin III plasma levels have been observed in sepsis [35]. HCC1 has been identified as a monocyte chemoattractant, with high concentrations observed in patients with chronic renal failure [36]. P-selectin is part of the selectin family of cell adhesion molecules, that promotes inflammatory reactions [37]. ADAMTS-13 is a metalloprotease with thrombospondin repeats, and has shown low activity in patients with recurrent thrombaotic thrombocytopenic purpura [38] [39]. In a previous study by Liu et al, GDF-15 levels were not significantly different between TB patients, latently infected individuals and healthy controls [33]. However, GDF-15 levels showed potential in the current study. In agreement with the findings of the current study, Mukae et al observed significantly higher levels of p-selectin in TB patients in comparison to individuals with ORD [40]. For the other newly identified TB diagnostic candidates, we observed higher levels of HCC1 and ADAMTS-13 in TB patients, but the levels of antithrombin III and NCAM were higher in individuals with ORD.

NCAM (CD56) is important in cell-cell or cell-matrix interactions, and is involved in neuronal differentiation, branching and survival [41]. It has been shown to play a role in lung tumor progression [42]. In the present study, we demonstrate for the first time, the potential of NCAM as a biomarker for TB disease. NCAM was the most frequently occurring marker in biosignatures for the diagnosis of TB disease, and was included in all the top 13 marker combinations for the diagnosis of TB disease regardless of HIV infection status, and in 68% of the models that were generated when HIV infected individuals were excluded. The combination of NCAM with five other markers (SAP, IL-1β, sCD40L, IL-13 and Apo A-1), diagnosed TB disease regardless of HIV infection status, with high accuracy, whereas all the TB patients and individuals with ORD were accurately classified (100% sensitivity and specificity) when NCAM was used in combination with either A2M+IL-22+ferritin+myoglobulin+IL-12(p40), or A2M+IL-22+ferritin+TNF-β+MIP-4, in the absence of HIV infection. In our previous serum-based study [11], optimal diagnosis of TB disease was achieved using a seven-marker biosignature of CRP, IFN-γ, IP-10, Apo A-1, transthyretin, SAA and CFH. While our previous study was conducted on 716 participants that were recruited from five different African countries, the current study was conducted on 55 individuals who were recruited at a single study site, and analysis was performed on plasma and not serum samples. There was excellent agreement between the findings of this and the serum study, as the diagnostic potential observed for individual host markers including CRP, SAA, SAP, CFH, Apo A-1, transthyretin, ferritin amongst others, was replicated in the current study. This implies that the new host markers identified in this study may be used in conjunction with, or as alternatives to the markers that were included in the previous seven-marker biosignature [11] if a point-of-care screening test based on these biosignatures were to be developed. However, further studies are required to determine the best combination of analytes before such a screening test can be developed.

In addition to being potentially useful as diagnostic candidates for TB disease, 11 of the markers investigated including CRP, SAP, ferritin, IP-10, α-1-antitrypsin changed with treatment, thereby indicating that they may be potential candidates for monitoring of the response to TB treatment. Although our observations for MMP-2 (increasing levels from baseline to month 6) were contrary to the observations of Ugarte-Gil et al (decreasing levels from baseline to month 6 in sputum culture positive individuals after 2 weeks of treatment) [43], our observations for CRP, SAP, ferritin, IP-10, and α-1-antitrypsin are in agreement with findings from previous studies [44] [45]. More studies are required to validate the potential of these biomarkers, and investigate whether any of the markers would be able to distinguish between clinical cure, treatment failure and relapse. Furthermore, it will be important that samples collected early after the initiation of TB treatment and regularly in the course of treatment are included in such future studies, as biomarkers for early indication of the response to TB treatment are an urgent need worldwide.

The main limitation of the current study was the small sample size. Inspite of this limitation, we confirmed the diagnostic potentials of host markers that were only recently identified as potential TB diagnostic candidates, identified new TB host biosignatures, and our study was done on individuals that presented with signs and symptoms, prior to the establishment of a clinical diagnosis, and at a community level health care clinic in a high-burden setting. Further studies are required to validate the new findings from our study preferably in larger numbers of study participants presenting with signs and symptoms requiring investigation for TB disease, and in studies conducted in other geographical regions. As the number of HIV infected study participants were limited in the current study, the biosignatures identified in this study require further investigation in HIV infected individuals. Importantly, HIV infected individuals included in such future studies should be staged with CD4 cell counts and viral loads so as to investigate the influence of severe HIV infection on the accuracy of the biosignatures. As we only investigated adult culture positive TB patients in the present study, the utility of the biosignatures also needs to be assessed in difficult to diagnose TB cases such as paediatric and extrapulmonary TB, smear and culture negative TB, and also in patients presenting with confirmed diseases that are similar to TB, including non-TB pneumonias. Validated biosignatures could then be incorporated into a point-of-care screening test for TB, preferably based on the lateral flow technology as recently demonstrated in a multi-centered African study [46].

In conclusion have identified candidate host markers, some of which have not previously been investigated in TB, as diagnostic candidates for TB disease. The biosignatures identified in our study require further validation in large-scale multi-site prospective studies.

## MATERIALS AND METHODS

### Study participants

Participants enrolled into the present study were individuals who presented with signs and symptoms requiring investigation for TB disease at the Fisantekraal Community Clinic in the outskirts of Cape Town, South Africa. The study was a sub-study of a larger diagnostic biomarker project (the African European Tuberculosis Consortium), that was ongoing at the study site and at field sites situated in six other African countries (www.ae-tbc.eu). All study participants presented with persistent cough lasting ≥ 2 weeks and at least one of either fever, malaise, recent weight loss, night sweats, knowledge of close contact with a TB patient, haemoptysis, chest pain or loss of appetite. Participants were eligible for the study if they were 18 years or older and willing to give written informed consent for participation in the study, including consent for HIV testing. Patients were excluded if they were pregnant, had not been residing in the study community for more than 3 months, were severely anaemic (haemoglobin < 10 g/l), were on anti-TB treatment, had received anti-TB treatment in the previous 90 days or if they were on quinolone or aminoglycoside antibiotics during the past 60 days. The study was approved by the Health Research Ethics Committee of the Faculty of Medicine and Health Sciences of the University of Stellenbosch.

### Sample collection and diagnostic tests

At enrolment, 6ml of blood was collected into heparinized BD vacutainer tubes (BD Biosciences, Franklin Lakes, NJ, USA) and transported to the laboratory at 4-8°C for further processing. Upon receipt in the laboratory, tubes were centrifuged at 2000 rpm for 10 minutes after which plasma was harvested, aliquoted and stored at −80°C until analysed. Sputum samples were collected from all study participants and cultured using the MGIT method (BD Biosciences). Positive MGIT cultures were examined for acid fast bacilli using the Ziehl-Neelsen technique (to check for contamination), followed by Capilia TB testing (TAUNS, Numazu, Japan), to confirm the isolation of organisms of the *M.TB* complex, before being designated as positive cultures.

### Classification of study participants and reference standard

As previously described, participants were classified as definite TB cases, probable TB cases, participants with other respiratory diseases (ORD) or questionable disease status using a combination of clinical, radiological, and laboratory findings [8]. However, only definite TB cases (culture positive individuals) and those with ORD were included in this discovery study. Briefly, individuals with ORD had a range of other diagnoses, including upper and lower respiratory tract infections (viral and bacterial infections, although attempts to identify organisms by bacterial or viral cultures were not made), and acute exacerbations of chronic obstructive pulmonary disease or asthma. Because of the disproportionately high number of individuals with ORD, we included all the 22 culture positive TB cases that were available at the study site and randomly selected 33 individuals with ORD from the study biobank, for inclusion into the current study.

### Luminex multiplex immunoassay

The concentrations of 74 host markers including alpha-2-macroglobulin (A2M), haptoglobin, C-reactive protein (CRP), serum amyloid P (SAP), procalcitonin (PCT), ferritin, tissue plasminogen activator (TPA), fibrinogen, serum amyloid A (SAA) (kits purchased from Bio-Rad Laboratories, Hercules, CA, USA), vitronectin, extracellular matrix protein 1 (ECM1), antithrombin III, vitamin D binding protein (VDBP), sFas, granzyme A, sFasL, sCD137, granzyme B, perforin, myoglobulin, ADAMTS13, P-selectin, lipocalin-2, growth differentiation factor (GDF) −15, thrombopoietin (TPO), stem cell factor (SCF), B-cell attracting chemokine (BCA)-1, epithelial neutrophil activating protein (ENA-78), thymic stromal lymphopoietin (TSLP), I-309(CCL-1), stromal cell derived factor-1 alpha (SDF-1α), IFN-γ, IFN-α2, interferon gamma inducible protein (IP)-10 (CXCL10), macrophage inflammatory protein (MIP)-1β, tumor necrosis factor (TNF)-α, TNF-β, vascular endothelial growth factor (VEGF), soluble CD40 ligand (sCD40L), apolipoprotein (Apo) A-1, Apo CIII, complement component 3 (CC3), transthyretin, complement factor H (CFH), total plasminogen activator inhibitor-1 (PAI-1), neural cell adhesion molecule (NCAM), brain-derived neurotrophic factor (BDNF), cathepsin D, myeloperoxidase (MPO), matrix metalloproteinase (MMP)-2, MMP-9, monokine induced by gamma interferon (MIG/CXCL9), granulocyte chemotactic protein-2 (GCP2), interferon inducible T-cell alpha chemoattractant (I-TAC/CXCL11), hemofiltrate CC chemokine-1 (HCC1), α1-antitrypsin, pigment epithelium derived factor (PEDF), macrophage inflammatory protein-4 (MIP-4/CCL18), complement C4, interleukin (IL)-17F, IL-17A, IL-22, IL-33, IL-21, IL-23, IL-25, IL-31, IL-28A, IL-16, IL-1β, IL-12(p40), IL-13, IL-11 and IL-29 (kits purchased from Merck Millipore, Billerica, MA, USA), were investigated in plasma samples from all the study participants. The experiments were performed blindly, according to the instructions of the kit manufacturers, on the Bio-Plex platform (Bio-Rad). The Bio-Plex manager Software version 6.1 was used for bead acquisition and analysis of median fluorescence intensities.

### Statistical analysis

Differences in the concentrations of host markers between TB patients and individuals with ORD were analysed using the Mann-Whitney U test. The diagnostic abilities of individual host markers were assessed by receiver operator characteristics (ROC) curve analysis. Optimal cut-off values and associated sensitivity and specificity were determined based on the Youden's Index [14]. The predictive abilities of combinations of host markers were investigated by general discriminant analysis (GDA), with leave-one-out cross validation [15]. Differences in the expression profiles of host markers during the course of TB treatment were analysed using mixed model repeated measures analysis of variance (ANOVA), with Fisher's Least Significant Difference (LSD) post hoc testing. P-values ≤ 0.05 were considered significant. The data were analysed using Statistica (Statsoft, Ohio, USA) and Graphpad Prism version 5 (Graphpad Software Inc., CA, USA).
